# Influence of American Society of Anesthesiologists Score on Oncologic Outcomes in Patients With Upper Tract Urothelial Carcinoma After Radical Nephroureterectomy: A Large-Sample Study in Two Institutions

**DOI:** 10.3389/fonc.2021.723669

**Published:** 2021-10-04

**Authors:** Yichu Yuan, Yiqiu Wang, Nan Zhang, Xiawa Mao, Yiran Huang, Jiwei Huang, Na Ji

**Affiliations:** ^1^ Department of Urology, Second Affiliated Hospital, Zhejiang University School of Medicine, Hangzhou, China; ^2^ Department of Urology, Renji Hospital, Shanghai Jiao Tong University School of Medicine, Shanghai, China; ^3^ Department of Anesthesia, Second Affiliated Hospital, Zhejiang University School of Medicine, Hangzhou, China

**Keywords:** radical nephroureterectomy, upper tract urothelial cancer, prognosis, survival, American Society of Anesthesiologists (ASA) score

## Abstract

**Introduction:**

As a research team of urologists and an anesthetist, we sought to investigate the prognostic significance of American Society of Anesthesiologists (ASA) score in patients with upper tract urothelial cancer (UTUC) after radical nephroureterectomy (RNU). ASA physical status (ASA-PS) classification not only was found to be associated with increased comorbidities but also independently factors for predicting morbidity and mortality. Accurate risk assessment was being particularly important for patients being considered for surgery.

**Methods:**

Records for 958 patients with UTUC who underwent RNU were reviewed. Clinicopathologic variables, including ASA-PS, were assessed at two institutions. Overall survival (OS), cancer-specific survival (CSS), intravesical recurrence-free survival (IRFS), and metastasis-free survival (MFS) were estimated using the Kaplan–Meier method and Cox regression analyses. We measured the independent predictive value of ASA-PS for mortality by multivariate regression. Association of ASA-PS and clinicopathologic variables was assessed.

**Results:**

The group of patients with ASA = 2/3 had a shorter 5-year OS (67.6% and 49.9%), CSS (72.9% and 58.1%), and MFS (75.1% and 58.5%). The median follow-up time was 39 months. Kaplan–Meier curves showed that the group with ASA = 2/3 had significantly poorer OS, CSS, and MFS. Adjusting for multiple potential confounding factors, multivariate analyses suggested that ASA score was an independent predictor of OS, CSS, and MFS (p = 0.004, p = 0.005, p < 0.001).

**Conclusion:**

Higher ASA scores were independently associated with lower survival rate. This capability, along with its simplicity, makes it a valuable prognostic metric. It should be seriously referenced in UTUC patients being considered for RNU.

## Introduction

Although radical nephroureterectomy (RNU) with bladder cuff excision is considered the gold standard for the treatment of upper tract urothelial cancer (UTUC), distant relapses are common for locally advanced high-grade disease. UTUC is the most aggressive malignant tumor of the urinary system (1); 30% of patients demonstrate invasive and/or locally advanced disease, 30%–40% have regional lymph node (LN) involvement, and 20% have metastatic disease (2, 3). The 5-year cancer-specific survival (CSS) ranging from 50% to 80% is for UTUC patients who underwent RNU (4, 5). It is of great significance to establish effective prediction methods to assist clinicians in making treatment decisions and follow-up strategies. For UTUC patients, pathological stage, tumor grade, lymph node metastasis, and tumor multifocality are known to be well-established prognostic factors (6–8). Potential prognostic preoperative factors would benefit outcome prediction and individual patient treatment choices.

American Society of Anesthesiologists physical status (ASA-PS) classification, as a standardized way for anesthesiologists to convey information about the patient’s overall health status, allows outcomes to be stratified by a global assessment. It was first introduced in 1940 and has been updated. Nowadays, ASA-PS classification includes a 6-point scale (**Table 1**). It has been demonstrated as a significant prognostic factor for the treatment outcome of bladder cancer (9), hepatocellular cancer (10), and endometrial cancer (11). Given the positive effects of the RNU on clinical benefits, it is reasonable to speculate that the magnitude and the impact of established morbidity predictors may be altered in UTUC patients undergoing RNU.

**Table 1:** American Society of Anesthesiologists (ASA) physical status classification.

ASA Score	Definition
ASA 1	A normal healthy patient
ASA 2	A patient with mild systemic disease
ASA 3	A patient with severe systemic disease
ASA 4	A patient with severe systemic disease that is a constant threat to life
ASA 5	A moribund patient who is not expected to survive without the operation
ASA 6	A declared brain-dead patient whose organs are being removed for donor purposes

For the purpose of investigating the influence of ASA scores on the long-term oncologic outcomes in patients diagnosed with UTUC, we set up several endpoints including overall survival (OS), CSS, intravesical recurrence-free survival (IRFS), and metastasis-free survival (MFS) and utilized the clinical data of 958 UTUC patients who underwent RNU in two Chinese institutions.

## Methods

### Patients

Patients with one of the following conditions were excluded: (1) incomplete clinical or pathological data; (2) underwent neoadjuvant chemotherapy or radiotherapy; (3) non-primary UTUC; (4) preoperative distant metastasis; (5) presence of other tumor types. Additionally, when computed tomography urography or other imaging examinations could not provide enough information to help clinicians make a definite diagnosis, diagnostic ureteroscopy with or without biopsy was used. In our study, 518 patients underwent diagnostic ureteroscopy. Information on 958 patients’ clinical and pathological features, including ASA score, gender, age, tumor location and size, smoking history, hydronephrosis, concomitant non-muscle invasive bladder cancer (NMIBC) or history of NMIBC, surgical methods, tumor grade and pathological stage, lymph node metastasis, tumor architecture and differentiation, lymphovascular invasion (LVI), multifocality and history of adjuvant chemotherapy (AC), was collected from Renji Hospital and Second Affiliated Hospital, Zhejiang University School of Medicine. This study was approved by the institutional review board, and approval number was 2020-369. Pathological stage was uniformly adjusted with reference to the 2017 TNM classification system (12). The histological grade was assessed according to the 2016 WHO consensus classification (13). Tumor multifocality was defined as the synchronous presence of multiple tumors in the renal pelvis or ureter. ASA score was used to assess the physical status of patients before RNU. Patients after surgery were followed up by telephone and outpatient.

### Follow-Up

The study endpoints were OS, CSS, IRFS, and MFS. According to the National Comprehensive Cancer Network (NCCN) Clinical Practice Guidelines, patients were assessed by computed tomography and/or magnetic resonance imaging to detect any findings suspected of disease progression every 3–4 months in the first year after surgery, every 6 months from the second through fifth year, and annually thereafter. In addition, history taking, physical examination, routine blood and serum chemistry lab work, urinary cytology, chest radiography, and cystoscopy were also included. OS was defined as the period from the date of surgery to patient death from any cause. CSS was defined as the time in months from date of surgery to cancer-related death. The cause of death was determined by the treating physicians and institutional cancer registries, by chart review corroborated by death certificates, or by death certificates alone. IRFS was defined as the time in months from the date of surgery to bladder recurrence. MFS was defined as the time in months from date of surgery to tumor metastasis.

### Statistical Method

Statistical tests were performed using SPSS version 22.0 (IBM Corp., Armonk, NY, USA). Proportions of the variables were analyzed using the chi-square test. Variables that had a univariate association with OS, CSS, IRFS, and MFS (p < 0.05) were included in the multiple Cox regression model. Hazard ratio (HR) and 95% CI were presented for selected items. Kaplan–Meier method with the log-rank test was used to assess OS, CSS, IRFS, and MFS. Multivariate analysis was conducted using Cox regression model to evaluate ASA-PS as an independent predictor of survival. All statistical tests were two-sided, and p < 0.05 was considered statistically significant.

## Results

### Baseline Clinicopathological Characteristics

Clinical and pathologic characteristics of 958 patients are shown in **Table 2**. There were 630 males (65.8%) and 328 females (34.2%), aged 30–89 years, with a median age of 67 years. Among them, 489 patients (51.1%) had UTUC located in the renal pelvis, 394 (41.1%) in the ureter, and 75 (7.8%) in both sites. A total of 252 patients (26.3%) had a history of smoking. Hydronephrosis was present in 499 patients (52.1%). In addition, 119 patients (12.4%) had synchronous presence or with a history of NMIBC. Open RNU and laparoscopic RNU were performed in 415 (43.3%) and 543 patients (56.7%), respectively. Here, 543 patients (51.6%) had a tumor ≤3 cm, and 415 (48.4%) had a tumor >3 cm. Low and high pathological grade was diagnosed in 275 (28.7%) and 683 patients (71.3%), respectively. The distribution of pathological stage was pT_a-1_ in 441 patients (46.0%), pT_2_ in 180 (18.8%), pT_3_ in 308 (32.2%), and pT_4_ in 29 (3.0%). Lymphadenectomy was performed in 227 patients (23.7%), and 62 (6.5%) were pathologically confirmed lymph node metastasis. Here, 516 patients (53.9%) showed papillary architecture, and 442 (46.1%) showed sessile architecture. Squamous or glandular differentiation and LVI were detected in 48 (5.0%) and 150 patients (15.7%), respectively. Moreover, 134 patients (14.0%) were multifocality. Of all patients, 196 patients received AC, including 53 pT_a-1_ patients and 143 pT_2–4_ patients. As to these 53 pT_a-1_ patients, we found most of them had the presence of risk factors, including positive lymph node, high tumor grade, tumor size >3 cm, and flat architecture.

**Table 2:** Clinicopathological characteristics of UTUC patients stratified by ASA scores.

Variable	All (N = 958)	ASA 1 (N = 167)	ASA 2 (N = 663)	ASA 3 (N = 128)	p	
Gender					0.408	
Female	328 (34.2)	63 (37.7)	218 (32.9)	47 (36.7)	
Male	630 (65.8)	104 (62.3)	445 (67.1)	81 (63.3)		
Age, years					<0.001*	
<65	383 (40.0)	101 (60.5)	259 (39.1)	23 (18.0)		
≥65	575 (60.0)	66 (39.5)	404 (60.9)	105 (82.0)		
Tumor location					0.598	
Renal pelvis	489 (51.1)	89 (53.3)	332 (50.1)	68 (53.1)		
Ureter	394 (41.1)	68 (40.7)	279 (42.1)	47 (36.7)		
Renal pelvis + Ureter	75 (7.8)	10 (6.0)	52 (7.8)	13 (10.2)		
Smoking					0.001*	
No	706 (73.7)	142 (85.0)	470 (71.0)	94 (73.4)		
Yes	252 (26.3)	25 (15.0)	193 (29.0)	34 (26.6)		
Hydronephrosis					0.295	
No	459 (47.9)	88 (52.7)	307 (46.3)	64 (50.0)		
Yes	499 (52.1)	79 (47.3)	356 (53.7)	64 (50.0)		
History of or with NMIBC					0.117	
No	839 (87.6)	149 (89.2)	585 (88.2)	105 (82.0)		
Yes	119 (12.4)	18 (10.8)	78 (11.8)	23 (18.0)		
Surgical methods					0.002*	
Laparoscopic RNU	543 (56.7)	96 (57.5)	393 (59.3)	54 (42.2)		
Open RNU	415 (43.3)	71 (42.5)	270 (40.7)	74 (57.8)		
Tumor size					0.014*	
≤3 cm	543 (51.6)	105 (62.9)	379 (57.2)	59 (46.1)		
>3 cm	415 (48.4)	62 (37.1)	284 (42.8)	69 (53.9)		
Tumor grade					0.753	
Low	275 (28.7)	44 (26.3)	193 (29.1)	38 (29.7)		
High	683 (71.3)	123 (73.7)	470 (70.9)	90 (70.3)		
Pathological stage					0.649	
pT_a-1_	441 (46.0)	79 (47.3)	299 (45.1)	63 (49.2)		
pT_2–4_	517 (54.0)	88 (52.7)	364 (54.9)	65 (50.8)		
Lymph node metastasis					0.918	
No	896 (93.5)	155 (92.8)	621 (93.7)	120 (93.8)		
Yes	62 (6.5)	12 (7.2)	42 (6.3)	8 (6.2)		
Tumor architecture					0.736	
Papillary	516 (53.9)	92 (55.1)	359 (54.1)	65 (50.8)		
Sessile	442 (46.1)	75 (44.9)	304 (45.9)	63 (49.2)		
Differentiation					0.613	
No	910 (95.0)	161 (96.4)	627 (94.6)	122 (95.3)		
Yes	48 (5.0)	6 (3.6)	36 (5.4)	6 (4.7)		
LVI					0.018*	
No	808 (84.3)	152 (91.0)	554 (83.6)	102 (79.7)		
Yes	150 (15.7)	15 (9.0)	109 (16.4)	26 (20.3)		
Multifocality					0.146	
No	824 (86.0)	149 (89.2)	571 (86.1)	104 (81.3)		
Yes	134 (14.0)	18 (10.8)	92 (13.9)	24 (18.7)		
Adjuvant chemotherapy					0.003*	
No	762 (79.5)	122 (73.1)	526 (79.3)	114 (89.1)		
Yes	196 (20.5)	45 (26.9)	137 (20.7)	14 (10.9)		

ASA, American Society of Anesthesiologists; RNU, radical nephroureterectomy; UTUC, upper tract urothelial cancer. *p < 0.05.

### Relationship Between ASA Scores and Clinical Characteristics

A total of 167 patients (17.4%) were classified as ASA score = 1, 663 (69.2%) as ASA score = 2, and 128 (13.4%) as ASA score = 3. There was no significant difference with regard to gender, hydronephrosis, tumor location, history of or with NMIBC, pathological stage, lymph node metastasis, tumor architecture, tumor grade, differentiation, and multifocality between different ASA scores (all p > 0.05). However, the distribution of characteristics was significantly varied in age (p < 0.001), smoking (p = 0.001), surgical methods (p = 0.002), tumor size (p = 0.014), LVI (p = 0.018), and AC (p = 0.003).

### Prognostic Significance of Survival

The median follow-up time was 39 months (ranged from 2 to 206 months). During the follow-up, a total of 304 patients (31.7%) died and 236 patients (24.6%) died from UTUC. A total of 225 patients (23.2%) had metastasis, including 96 (42.7%) with lung metastases, 52 (23.1%) with bone metastases, 42 (18.7%) with liver metastases, 15 (6.7%) with lymph node metastasis, nine (4.0%) with lumbar muscle metastases, seven (3.1%) with posterior peritoneal metastasis, and four (1.7%) in other sites (**Figure S1**).

**Figure 1:**
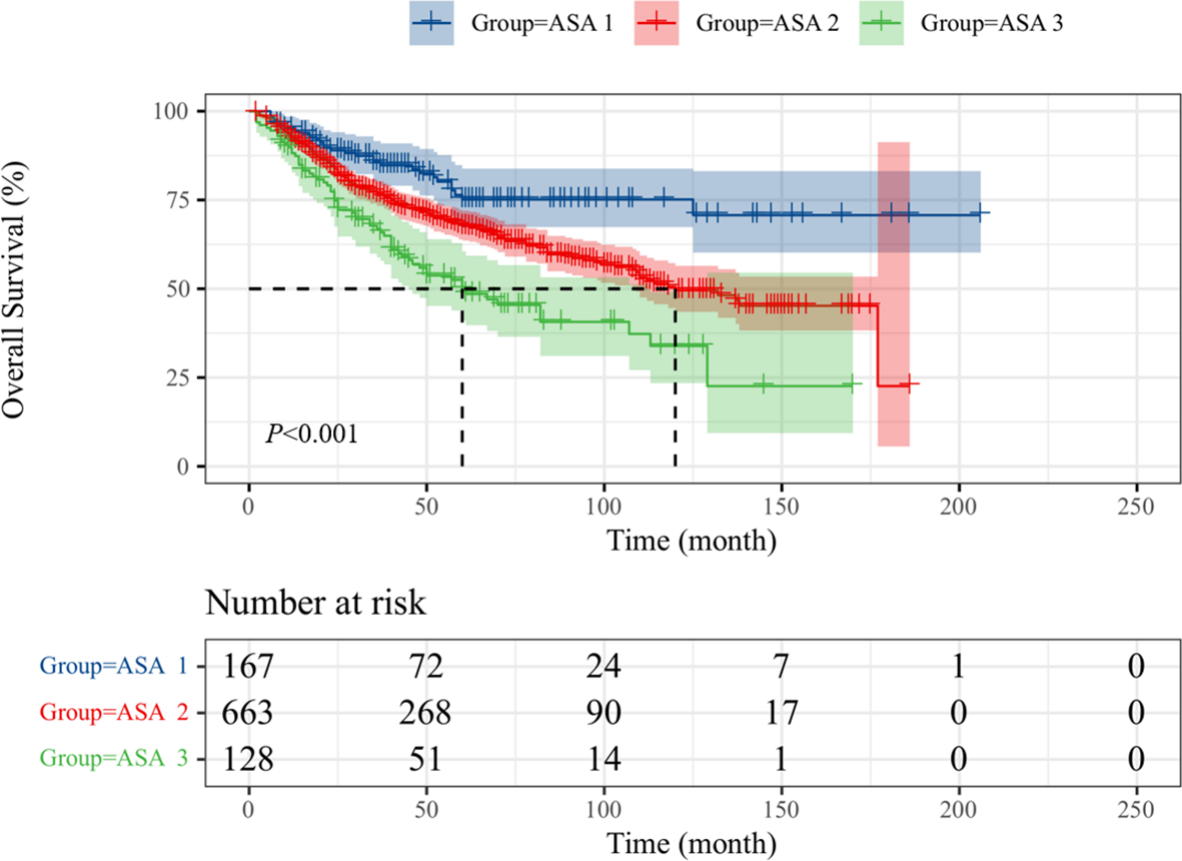
Kaplan-Meier curves for OS stratified according to ASA scores.

**Figure 2:**
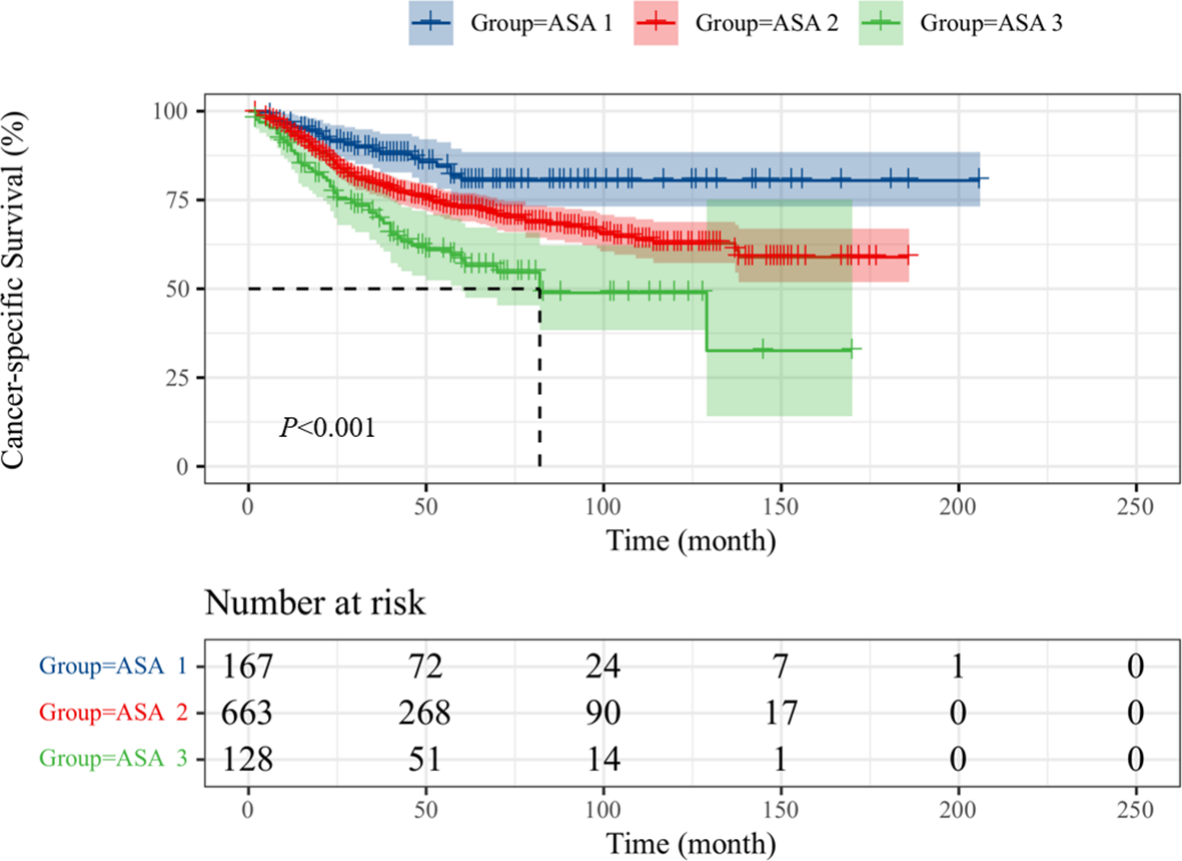
Kaplan-Meier curves for CSS stratified according to ASA scores.

**Figure 3:**
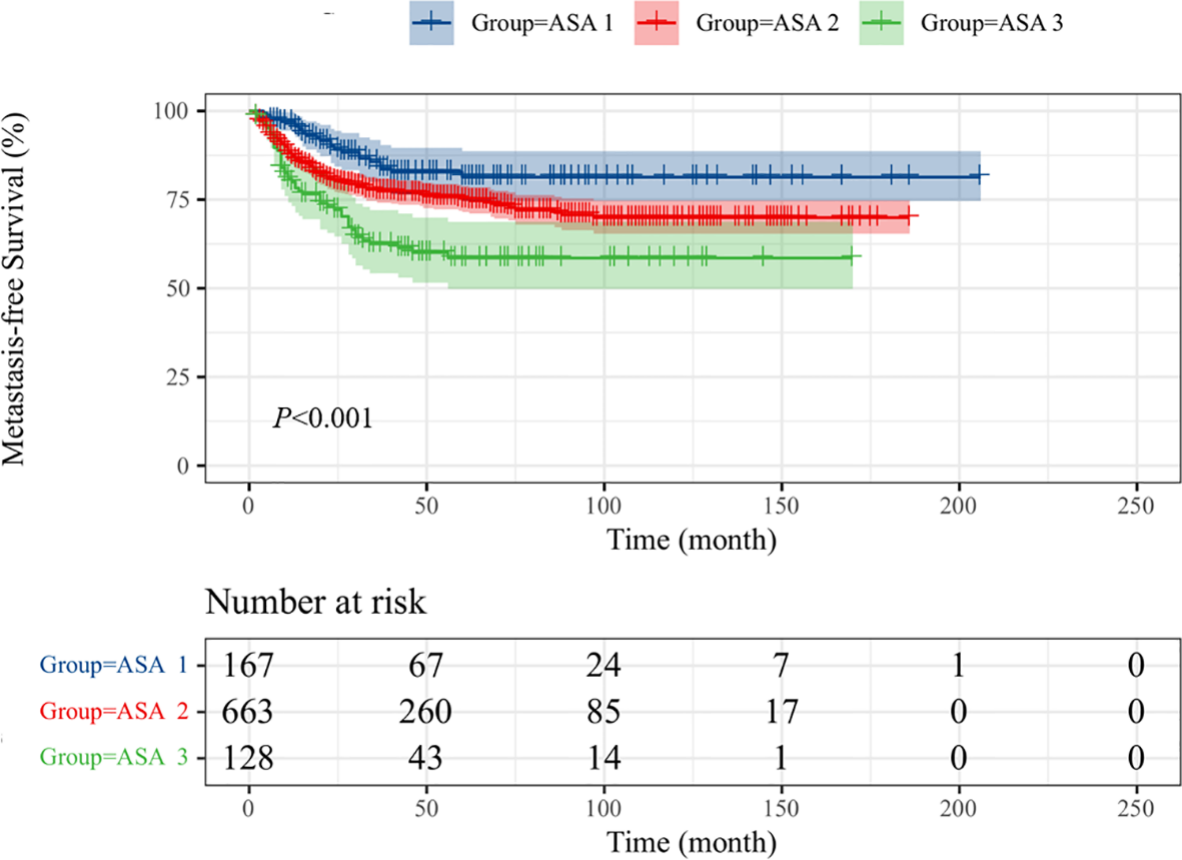
Kaplan-Meier curves for MFS stratified according to ASA scores.

**Figure 4:**
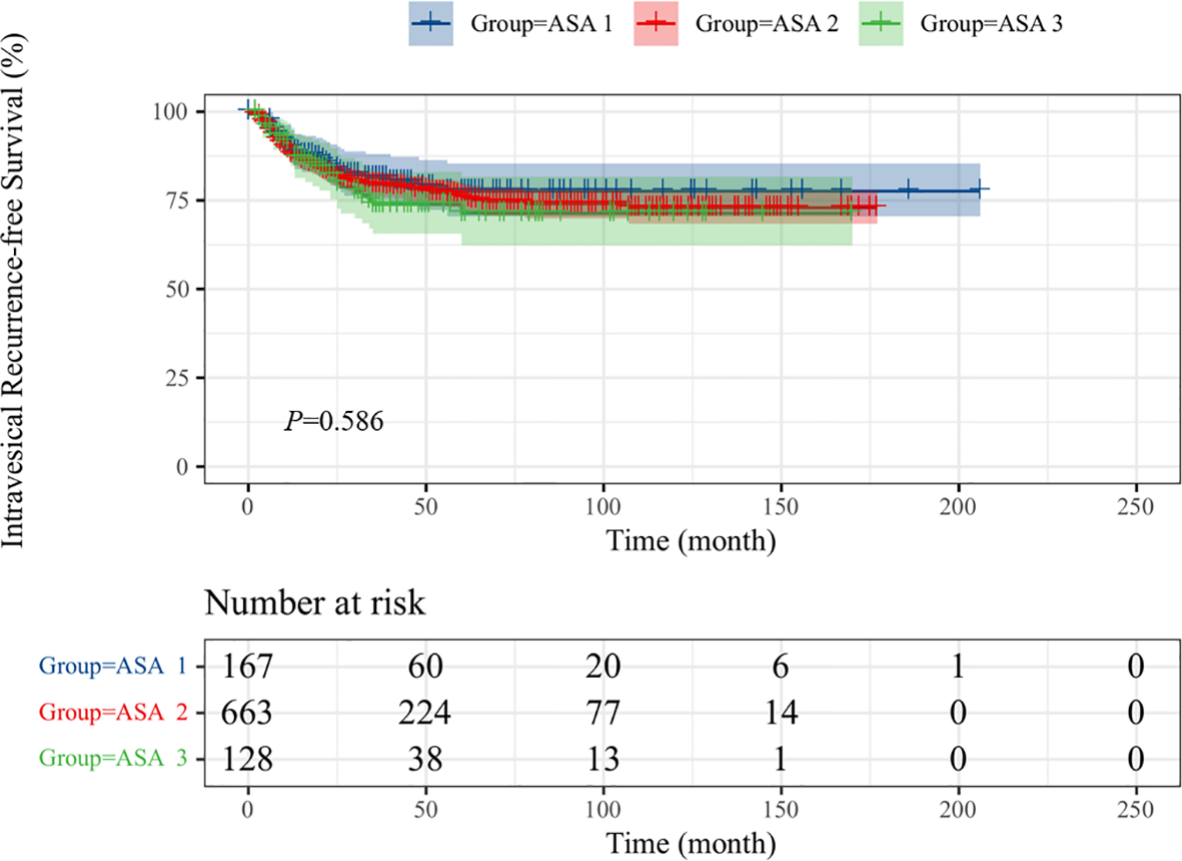
Kaplan-Meier curves for IRFS stratified according to ASA scores.

Intravesical recurrence and distant metastasis occurred in 192 (20.0%) and 225 patients (23.5%), respectively. To explore the prognostic significance of ASA scores in UTUC, Kaplan–Meier survival curves were generated, and groups were compared using the log-rank test. Patients with low ASA score (ASA = 1) had a significantly reduced rate of survival than those with high ASA score (ASA = 2/3) with regard to OS (**Figure 1**; p < 0.001), CSS (**Figure 2**; p < 0.001), and MFS (**Figure 3**; p < 0.001), but not IRFS (**Figure 4**; p = 0.586). The 5-year OS rates after RNU were 75.2% for the ASA = 1 group, 67.6% for the ASA = 2 group, and 49.9% for the ASA = 3 group. The 5-year CSS rate was 80.5% in patients with ASA = 1, 72.9% in patients with ASA = 2, and 58.1% in patients with ASA = 3. As to 5-year MFS rate, ASA = 1/2/3 group had 81.3%, 75.1%, and 58.5%, respectively. However, no significant differences in IRFS were observed among the different ASA scores.

Cox regression analyses were performed with ASA-PS score as reference. All-cause mortality and cancer-related mortality were increased in patients whose score was 3 or 2.

The univariate analysis also revealed that age ≥65 years, tumor location, hydronephrosis, history of or with NMIBC, tumor size, higher tumor grade and pathological stage, sessile tumor, lymph node metastasis, differentiation, LVI, and multifocality were significantly associated with OS, CSS, and MFS. Besides, AC was significantly associated with CSS/MFS but not OS.

A multivariate model was constructed to identify the factors associated with OS (**Table 3**) and CSS (**Table 4**). In multivariate analysis, ASA = 2 and ASA = 3 [hazard ratio (HR) = 1.535, p = 0.030; HR = 2.133, p = 0.001] were independent predictors of OS, along with age ≥65 years (HR = 1.869, p < 0.001), history of or with NMIBC (HR = 1.740, p < 0.001), high grade (HR = 1.665, p = 0.001), pT_2–4_ (HR = 1.585, p = 0.001), lymph node metastasis (HR = 1.898, p = 0.001), sessile tumor (HR = 1.360, p = 0.027), and differentiation (HR = 1.728, p = 0.009).

**Table 3:** Univariable and multivariable Cox regression models to predict OS.

Parameters	Univariable Analysis		Multivariable Analysis	
	HR (95% CI)	p	HR (95% CI)	p
ASA score	–	<0.001*	–	0.004*
ASA 2/ASA 1	1.815 (1.244~2.649)	0.002*	1.535 (1.044~2.258)	0.030*
ASA 3/ASA 1	3.020 (1.964~4.643)	<0.001*	2.133 (1.354~3.361)	0.001*
Gender (Male/Female)	1.031 (0.812~1.309)	0.800	–	–
Age (≥65/<65 years)	2.102 (1.634~2.704)	<0.001*	1.869 (1.435~2.435)	<0.001*
Tumor location	–	<0.001*	–	0.232
Ureter/Renal	1.181 (0.928~1.504)	0.177	0.818 (0.605~1.105)	0.191
Renal Pelvis + Ureter/Renal Pelvis	2.243 (1.566~3.212)	<0.001*	1.340 (0.688~1.835)	1.123
Smoking (Yes/No)	1.057 (0.786~1.422)	0.713	–	–
Hydronephrosis (Yes/No)	1.377 (1.097~1.728)	0.006*	1.148 (0.869~1.516)	0.332
History of or with NMIBC (Yes/No)	2.507 (1.558~2.715)	<0.001*	1.740 (1.289~2.351)	<0.001*
Surgical methods (LNU/ONU)	1.129 (0.894~1.427)	0.309	–	–
Tumor size (>3 cm/≤3 cm)	1.419 (1.133~1.777)	0.002*	1.105 (0.872~1.399)	0.409
Tumor grade (High/Low)	2.423 (1.828~3.211)	<0.001*	1.665 (1.218~2.276)	0.001*
Pathological stage (pT2–4/pTa-1)	2.516 (1.972~3.210)	<0.001*	1.585 (1.194~2.103)	0.001*
Lymph node metastasis (Yes/No)	2.898 (2.024~4.149)	<0.001*	1.898 (1.294~2.778)	0.001*
Tumor architecture (Sessile/Papillary)	2.174 (1.726~2.738)	<0.001*	1.360 (1.036~1.786)	0.027*
Differentiation (Yes/No)	3.341 (2.314~4.824)	<0.001*	1.728 (1.147~2.602)	0.009*
LVI (Yes/No)	2.203 (1.699~2.857)	<0.001*	1.219 (0.912~1.629)	0.182
Multifocality (Yes/No)	1.651 (1.249~2.183)	<0.001*	1.191 (0.819~1.732)	0.361
Adjuvant chemotherapy (No/Yes)	1.257 (0.954~1.655)	0.104	–	–

ASA, American Society of Anesthesiologists; HR, hazard ratio; LNU, laparoscopic nephroureterectomy; LVI, lymphovascular invasion; NMIBC, non-muscle invasive bladder cancer; ONU, open nephroureterectomy; OS, overall survival. *p < 0.05.

**Table 4:** Univariable and multivariable Cox regression models to predict CSS.

Parameters	Univariable Analysis		Multivariable Analysis	
	HR (95% CI)	p	HR (95% CI)	p
ASA score	–	<0.001*	–	0.005*
ASA 2/ASA 1	1.863 (1.204~2.884)	0.005*	1.594 (1.020~2.490)	0.041*
ASA 3/ASA 1	3.144 (1.921~5.145)	<0.001*	2.347 (1.390~3.962)	0.001*
Gender (Male/Female)	1.016 (0.777~1.330)	0.905	–	–
Age (≥65/<65 years)	1.808 (1.370~2.386)	<0.001*	1.660 (1.230~2.241)	0.001*
Tumor location	–	<0.001*	–	0.323
Ureter/Renal	1.222 (0.928~1.610)	0.153	0.829 (0.588~1.169)	0.285
Renal Pelvis + Ureter/Renal Pelvis	2.459 (1.665~3.654)	<0.001*	1.153 (0.666~1.994)	0.611
Smoking (Yes/No)	1.000 (0.724~1.382)	0.999	–	–
Hydronephrosis (Yes/No)	1.446 (1.117~1.872)	0.005*	1.134 (0.828~1.555)	0.433
History of or with NMIBC (Yes/No)	2.150 (1.577~2.932)	<0.001*	1.830 (1.309~2.560)	<0.001*
Surgical methods (LNU/ONU)	1.057 (0.814~1.374)	0.675	–	–
Tumor size (>3 cm/≤3 cm)	1.667 (1.290~2.154)	<0.001*	1.280 (0.978~1.676)	0.073
Tumor grade (High/Low)	3.425 (2.387~4.914)	<0.001*	2.209 (1.492~3.270)	<0.001*
Pathological stage (pT_2–4_/pT_a-1_)	2.989 (2.244~3.982)	<0.001*	1.665 (1.194~2.321)	0.003*
Lymph node metastasis (Yes/No)	2.959 (1.992~4.405)	<0.001*	1.555 (1.013~2.387)	0.043*
Tumor architecture (Sessile/Papillary)	2.533 (1.938~3.309)	<0.001*	1.383 (1.014~1.886)	0.041*
Differentiation (Yes/No)	3.714 (2.500~5.517)	<0.001*	1.654 (1.065~2.569)	0.025*
LVI (Yes/No)	2.585 (1.948~3.431)	<0.001*	1.377 (1.005~1.886)	0.046*
Multifocality (Yes/No)	1.757 (1.289~2.396)	<0.001*	1.182 (0.778~1.797)	0.433
Adjuvant chemotherapy (No/Yes)	1.560 (1.164~2.089)	0.003*	1.380 (1.004~1.898)	0.047*

ASA, American Society of Anesthesiologists; HR, hazard ratio; LNU, laparoscopic nephroureterectomy; LVI, lymphovascular invasion; NMIBC, non-muscle invasive bladder cancer; ONU, open nephroureterectomy; CSS, cancer-specific survival. *p < 0.05.

As to CSS, multivariable analysis also demonstrated that ASA = 2 and ASA = 3 (HR = 1.594, p = 0.041; HR = 2.347, p = 0.001) were independent predictors, along with age ≥65 years (HR = 1.660, p = 0.001), history of or with NMIBC (HR = 1.830, p < 0.001), high grade (HR = 2.209, p < 0.001), pT_2–4_ (HR = 1.665, p = 0.003), lymph node metastasis (HR = 1.555, p = 0.043), sessile tumor (HR = 1.383, p = 0.041), differentiation (HR = 1.654, p = 0.025), LVI (HR = 1.377, p = 0.046), and AC (HR = 1.380, p = 0.047).

Adjusting for multiple potential confounding factors, ASA = 2 (HR = 1.706, p = 0.017) and ASA = 3 (HR = 2.859, p < 0.001) remained independently associated with decreased MFS (**Table 5**). In addition, history of or with NMIBC (HR = 1.566, p = 0.013), high tumor grade (HR = 2.523, p < 0.001), pT_2–4_ (HR = 2.158, p < 0.001), lymph node metastasis (HR = 1.730, p = 0.009), and AC (HR = 1.651, p = 0.001) were independent prognostic factors.

**Table 5:** Univariable and multivariable Cox regression models to predict MFS.

Parameters	Univariable Analysis		Multivariable Analysis	
	HR (95% CI)	p	HR (95% CI)	p
ASA score	–	<0.001*	–	<0.001*
ASA 2/ASA 1	1.716 (1.116~2.639)	0.014*	1.706 (1.099~2.649)	0.017*
ASA 3/ASA 1	2.857 (1.747~4.672)	<0.001*	2.859 (1.692~4.831)	<0.001*
Gender (Male/Female)	0.855 (0.653~1.119)	0.254	–	–
Age (≥65/<65 years)	1.446 (1.098~1.905)	0.009*	1.309 (0.972~1.762)	0.076
Tumor location	–	0.065	–	–
Ureter/Renal	1.063 (0.805~1.405)	0.666	–	–
Renal Pelvis + Ureter/Renal Pelvis	1.676 (1.085~2.589)	0.020*	–	–
Smoking (Yes/No)	1.119 (0.824~1.519)	0.472	–	–
Hydronephrosis (Yes/No)	1.569 (1.202~2.050)	0.001*	1.120 (0.851~1.473)	0.420
History of or with NMIBC (Yes/No)	1.787 (1.278~2.500)	0.001*	1.566 (1.099~2.230)	0.013*
Surgical methods (LNU/ONU)	1.168 (0.896~1.523)	0.251	–	–
Tumor size (>3 cm/≤3 cm)	1.519 (1.169~1.972)	0.002*	1.233 (0.943~1.613)	0.126
Tumor grade (High/Low)	4.170 (2.767~6.283)	<0.001*	2.523 (1.623~3.922)	<0.001*
Pathological stage (pT_2–4_/pT_a-1_)	3.772 (2.758~5.159)	<0.001*	2.158 (1.506~3.092)	<0.001*
Lymph node metastasis (Yes/No)	3.322 (2.257~4.878)	<0.001*	1.730 (1.147~2.604)	0.009*
Tumor architecture (Sessile/Papillary)	2.492 (1.896~3.275)	<0.001*	1.143 (0.836~1.563)	0.401
Differentiation (Yes/No)	3.181 (2.081~4.863)	<0.001*	1.454 (0.914~2.314)	0.114
LVI (Yes/No)	2.413 (1.798~3.238)	<0.001*	1.281 (0.928~1.767)	0.133
Multifocality (Yes/No)	1.589 (1.146~2.202)	0.005*	1.215 (0.863~1.712)	0.265
Adjuvant chemotherapy (No/Yes)	1.969 (1.483~2.615)	<0.001*	1.651 (1.212~2.251)	0.001*

ASA, American Society of Anesthesiologists; HR, hazard ratio; LNU, laparoscopic nephroureterectomy; LVI, lymphovascular invasion; MFS, metastasis-free survival; NMIBC, non-muscle invasive bladder cancer; ONU, open nephroureterectomy. *p < 0.05.

However, no significant difference was found in patients with different ASA scores in IRFS (**Table 6**). History of or with NMIBC (HR = 1.576, p = 0.022) and tumor size (HR = 1.379, p = 0.03) showed a significant effect on patients in the multivariate analysis.

**Table 6:** Univariable and multivariable Cox regression models to predict IRFS.

Parameters	Univariable Analysis		Multivariable Analysis	
	HR (95% CI)	p	HR (95% CI)	p
ASA score	–	0.586	–	–
ASA 2/ASA 1	1.174 (0.790~1.744)	0.428	–	–
ASA 3/ASA 1	1.305 (0.780~2.185)	0.311	–	–
Gender (Male/Female)	1.361 (0.994~1.864)	0.055	–	–
Age (≥65/<65 years)	1.180 (0.883~1.578)	0.263	–	–
Tumor location	–	0.002*	–	0.176
Ureter/Renal	1.262 (0.932~1.708)	0.132	1.230 (0.899~1.685)	0.196
Renal Pelvis + Ureter/Renal Pelvis	2.258 (1.434~3.555)	<0.001*	1.658 (0.930~2.955)	0.086
Smoking (Yes/No)	1.026 (0.734~1.435)	0.881	–	–
Hydronephrosis (Yes/No)	1.093 (0.823~1.452)	0.537	–	–
History of or with NMIBC (Yes/No)	1.947 (1.360~2.787)	<0.001*	1.576 (1.068~2.326)	0.022*
Surgical methods (LNU/ONU)	1.105 (0.829~1.473)	0.494	–	–
Tumor size (>3 cm/≤3 cm)	1.400 (1.055~1.859)	0.020*	1.379 (1.032~1.843)	0.030*
Tumor grade (High/Low)	1.455 (1.053~2.012)	0.023*	1.342 (0.965~1.865)	0.080
Pathological stage (pT_2–4_/pT_a-1_)	1.092 (0.822~1.451)	0.543	–	–
Lymph node metastasis (Yes/No)	1.410 (0.802~2.475)	0.233	–	–
Tumor architecture (Sessile/Papillary)	1.089 (0.817~1.451)	0.561	–	–
Differentiation (Yes/No)	1.375 (0.727~2.601)	0.327	–	–
LVI (Yes/No)	1.002 (0.667~1.505)	0.993	–	–
Multifocality (Yes/No)	1.657 (1.162~2.364)	0.005*	1.134 (0.717~1.792)	0.591
Adjuvant chemotherapy (No/Yes)	1.420 (1.021~1.975)	0.037*	1.296 (0.928~1.809)	0.128

ASA, American Society of Anesthesiologists; HR, hazard ratio; IRFS, intravesical recurrence-free survival; LNU, laparoscopic nephroureterectomy; LVI, lymphovascular invasion; NMIBC, non-muscle invasive bladder cancer; ONU, open nephroureterectomy. *p < 0.05.

## Discussion

Together with Adult Comorbidity Evaluation-27 (ACE-27), Charlson Comorbidity Index (CCI), and Eastern Cooperative Oncology Group (ECOG) performance status, the ASA-PS was one of the most commonly used comorbidity indices in the literature (14, 15).

In contrast to the ASA score, however, an anesthetist does not need to complete the CCI in practice schedule. Recently, some researchers aimed to quantify the relationship between the CCI and the ASA grade; the former was determined from documented International Classification of Diseases (ICD) codes, and the latter was assigned by the anesthetist. They have found that the addition of the demographic variables made for a much better predictive model and helped to explain considerably more of the variance in ASA grade than did CCI alone (16). That means in the real life of assigning an ASA grade, anesthetists would take full factors into account, not only the medical condition but also factors that may adversely influence a patient’s tolerance to an operative procedure. Such characteristics as well-documented data, such as age, body mass index (BMI), and lifestyle factors like smoking history, but perhaps also alcohol consumption and sometimes even the complexity of the planned surgery (17). ASA score was much more comprehensive than we might expect. It is also reported that the ASA score is superior to other notable scoring systems including the Charlson score (18) in predicting surgical outcomes. Admittedly, for UTUC, two systems ought to be further compared in randomized controlled trials. Unlike the results of our study, the 2021 European Association of Urology (EAU) Guidelines of muscle-invasive and metastatic bladder cancer (19) strongly recommend to “Assess comorbidity by a validated score, such as the Charlson Comorbidity Index. The American Society of Anesthesiologists score should not be used in this setting”.

ASA-PS has been demonstrated as a strong independent factor associated with postoperative morbidity and mortality (11, 20). Based on a large cohort of 6,301 patients who received surgery, Wolters et al. (21) have reported similar findings and found that high ASA score reflected delayed wound repair. Several studies have revealed that high ASA score was associated with prognosis in several urologic malignancies (9, 18, 22); limited evidence has shown that high ASA score was related to unfavorable prognosis in UTUC patients. Our results suggested that ASA score ≥2 was independently associated with poorer OS, CSS, and MFS, adjusting for a number of potential confounding variables. However, there was no significant difference with regard to IRFS between low and high ASA score groups in UTUC patients. This might indicate that ASA scores, as a general systemic status indicator, could not reflect localized disease completely. Additionally, we demonstrated the distinct relationship between ASA score and some clinicopathological characteristics, such as age, tumor size, LVI, and AC. Most of these indicators certainly had significant impact on the prognosis of UTUC patients (23–26).

Our results also showed that advanced age, high tumor grade and pathological stage, lymph node metastasis, sessile architecture, and differentiation were associated with worse OS and CSS, which was consistent with the results reported by Margulis et al. (27). Although previous studies have reported the prognostic value of ASA scores in UTUC patients (28, 29), our study is unique for several reasons. Primarily, in the present study, a total of 17 clinicopathological characteristics were analyzed, while a strong predictor of lymph node metastasis was not included in the study by Ho et al. (28) and only seven characteristics were collected in the study by Alexis et al. (29). Nearly twice as many in previous studies, a larger cohort had longer follow-up, and approximately 1,000 patients were retrospectively analyzed. Furthermore, given that survival is a heterogeneous endpoint, overall, cancer-specific, intravesical recurrence-free, and metastasis-free survival have all been involved.

Considering the underlying systemic disease with predisposition to poor recovery, infectious and cardiorespiratory complications were more common in patients with a high ASA score than in those with a low score. General condition and systemic illness were absolute barriers for systemic chemotherapy and also significant determinants of survival, as for ASA-PS, ranging from a healthy person (class 1) to one with a bad/severe systemic disorder that is a constant threat to life (30). When the cardiopulmonary system is not strong and working inefficiently, oxygen will not circulate all around the body helping to feed and renew our body tissues and vital organs (liver, kidney, et al.). Especially, there is additional task for tumor-host’s body to activate the antitumor effect, such as immunological function response. Different from other tumor types, renal insufficiency was an unavoidable problem for UTUC patients who underwent RNU. Renal insufficiency often constrains the choice of nephrotoxic chemotherapy regimen, which seems to be a challenge preventing the effective delivery of cisplatin-based chemotherapy (30–32). In this study, similarly, as ASA score increased, the proportion of AC has also risen (10.9%, 20.7%, and 26.9%, respectively, p = 0.003*; **Table 2**). It turns out that higher ASA score results in damage to the tumor-bearing host and tolerance to systemic therapy. In other words, disorder of the immune function will decrease the antitumor ability and simultaneously increase tumor burden (33).

Additionally, preoperative hydronephrosis and multifocality were considered to be correlated with adverse prognosis (8, 34); in contrast, our results found that these two indicators were not independent predictors. Our study reveals that hydronephrosis and multifocality do not appear to have a significant influence on survival outcome; this result has also been confirmed by previous studies (8, 35). We need to acknowledge a selection bias for exclusion of patients receiving AC. Given their impaired renal function, patients with hydronephrosis are more likely to miss the opportunity to receive AC after radical nephroureterectomy (36). The prognostic impact of multifocal UTUC has also been poorly understood. The ability of chemotherapy of eradicating micrometastasis has been widely recognized (37). Thomas et al. (8) have reported that tumor multifocality was not an independent predictor of both disease progression (HR = 1.43, p = 0.019) and CSS (HR = 1.46, p = 0.027) in advanced UTUC (stratified by non-confined type, the confounding effect of AC cannot be adequately adjusted).

There were several limitations associated with this study. Firstly, a retrospective analysis may cause a selection bias. Secondly, potential predictors including BMI, history of aristolochic acid, and preoperative renal function were not available. Thirdly, lymphadenectomy was not routinely performed and the extent of lymph node dissection was not standardized because the pattern and benefits of lymphadenectomy were still controversial. Moreover, as widely acknowledged, surgical patients with higher ASA score developed substantially higher rates of perioperative medical complications (20). Unfortunately, we do not know the detailed information of patients who have suffered perioperative complications. It was really a huge effort for researchers from two institutions to collect undocumented clinical data for almost a thousand patients. This aspect was an unavoidable limitation in the current study but could be improved by further study. Based on a large number of cases, the detailed clinical and pathological data with long-term follow-up of UTUC patients from two institutions in China, this study was able to provide more convincing information to clinicians.

## Conclusion

We suggest that RNU is safe for selected patients with UTUC. However, higher ASA score predicts poor clinical outcomes, and it was a significant prognostic factor for OS, CSS, and MFS. This prognostic factor may be a useful variable to include into future risk prediction and contribute to clinical decision-making. Long-term and larger-scale studies may produce more reliable results.

## Data Availability Statement

The raw data supporting the conclusions of this article will be made available by the authors without undue reservation.

## Ethics Statement

This study was approved by institutional review board, and approval number was 2020-369.

## Author Contributions

YY and YW contributed to the conceptualization, methodology, validation, visualization, formal analysis, investigation, and writing the original draft. NZ contributed to the conceptualization, resources, and funding acquisition. XM contributed to the methodology, visualization, software, and validation. YH contributed to the methodology, visualization, and software. JH and NJ contributed to the methodology, data curation, validation, investigation, resources, and writing–review and editing. All authors contributed to the article and approved the submitted version.

## Funding

This work was supported financially by Zhejiang Provincial Natural Science and Technology Department Public Welfare Projects (grant number LGC21H050001).

## Conflict of Interest

The authors declare that the research was conducted in the absence of any commercial or financial relationships that could be construed as a potential conflict of interest.

## Publisher’s Note

All claims expressed in this article are solely those of the authors and do not necessarily represent those of their affiliated organizations, or those of the publisher, the editors and the reviewers. Any product that may be evaluated in this article, or claim that may be made by its manufacturer, is not guaranteed or endorsed by the publisher.
