# Supplementary figures and images for: Influence of American Society of Anesthesiologists Score on Oncologic Outcomes in Patients With Upper Tract Urothelial Carcinoma After Radical Nephroureterectomy: A Large-Sample Study in Two Institutions
Source: Front Oncol. 2021 Oct 4;11:723669. doi: 10.3389/fonc.2021.723669 (PMC8521060; doi:10.3389/fonc.2021.723669)

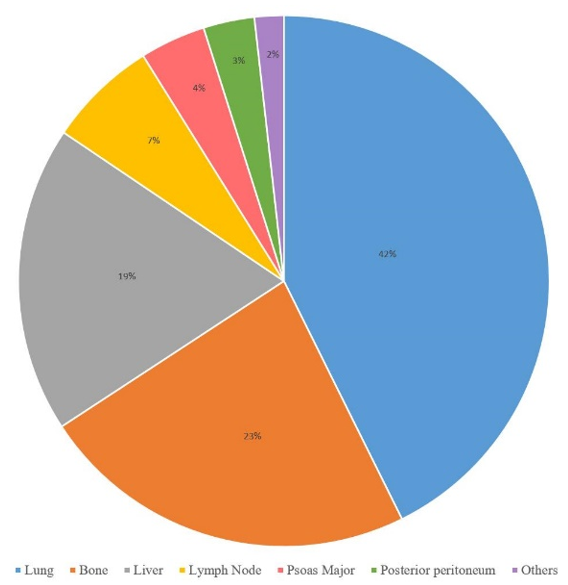


Fig S1. The distribution of postoperative metastatic sites of UTUC patients

Supplement: Supplementary file 1 [file DataSheet_1.docx]
